# Cognitive training reorganizes lateralization of fronto-parietal network in vascular cognitive impairment

**DOI:** 10.1093/braincomms/fcaf394

**Published:** 2025-10-10

**Authors:** Xinhu Jin, Yi Xing, Baihan Lyu, Junhua Ding, Xiuyi Wang, Yi Du, Yi Tang

**Affiliations:** Institute of Psychology, Chinese Academy of Sciences, Beijing 100101, China; State Key Laboratory of Cognitive Science and Mental Health, Institute of Psychology, Chinese Academy of Sciences, Beijing 100101, China; Department of Neurology, Xuanwu Hospital, Capital Medical University, Beijing 100053, China; Innovation Center for Neurological Disorders, Xuanwu Hospital, Capital Medical University, Beijing 100053, China; Key Laboratory of Neurodegenerative Diseases, Ministry of Education of the People’s Republic of China, Beijing 100053, China; Institute of Psychology, Chinese Academy of Sciences, Beijing 100101, China; Department of Psychology, University of Chinese Academy of Sciences, Beijing 100049, China; Department of Psychology, University of Edinburgh, Edinburgh EH8 9JZ, UK; Institute of Psychology, Chinese Academy of Sciences, Beijing 100101, China; State Key Laboratory of Cognitive Science and Mental Health, Institute of Psychology, Chinese Academy of Sciences, Beijing 100101, China; Institute of Psychology, Chinese Academy of Sciences, Beijing 100101, China; State Key Laboratory of Cognitive Science and Mental Health, Institute of Psychology, Chinese Academy of Sciences, Beijing 100101, China; Department of Psychology, University of Chinese Academy of Sciences, Beijing 100049, China; Chinese Institute for Brain Research, Beijing 102206, China; Department of Neurology, Xuanwu Hospital, Capital Medical University, Beijing 100053, China; Innovation Center for Neurological Disorders, Xuanwu Hospital, Capital Medical University, Beijing 100053, China; Key Laboratory of Neurodegenerative Diseases, Ministry of Education of the People’s Republic of China, Beijing 100053, China

**Keywords:** vascular cognitive impairment no dementia, computerized cognitive training, functional lateralization, resting-state fMRI, compensation by reorganization

## Abstract

Vascular cognitive impairment no dementia (VCIND) represents cognitive deficits due to vascular causes, without meeting the criteria for dementia. Cognitive training has emerged as a safe and effective intervention for VCIND, though its underlying mechanisms remain obscure. This study investigates how subcortical VCIND and computerized cognitive training affect brain functional lateralization of the fronto-parietal network (FPN), whose functions are notably influenced by both VCIND and cognitive training. In a randomized, active-controlled design for VCIND patients, we assessed the resting-state functional lateralization index (LI) of the FPN and conducted neuropsychological assessments in VCIND training and control groups (*n* = 30 per group) at baseline, after a 7-week intervention and at a 6-month follow-up. A healthy older group (*n* = 30) only provided baseline data. At baseline, VCIND patients showed an FPN lateralization pattern similar to that of healthy older adults. However, a stronger right-lateralized interhemispheric heterotopic LI in FPN correlated with better memory performance only in healthy adults. After the intervention, only the VCIND training group exhibited reduced lateralization in FPN, shifting to a bilateral interhemispheric LI, with stronger leftward changes correlating with improved executive and memory functions. Notably, these changes disappeared at the 6-month follow-up. These findings suggest that subcortical VCIND modifies the relationship between FPN lateralization and cognitive functions, rather than altering the lateralization pattern itself. Short-term computerized cognitive training facilitates executive and memory functions by promoting hemispherical reorganizing of FPN and functional compensation, although the benefits may diminish over time.

## Teaser

Xinhu *et al*. reported that healthy older adults and patients with subcortical vascular cognitive impairment no dementia use different coping strategies and distinct functional lateralization patterns of fronto-parietal network to support memory and executive function. This may inform cognitive training intervention to help prevent Alzheimer’s disease and other neurodegenerative diseases.

## Graphical Abstract

**Graphical Abstract:**
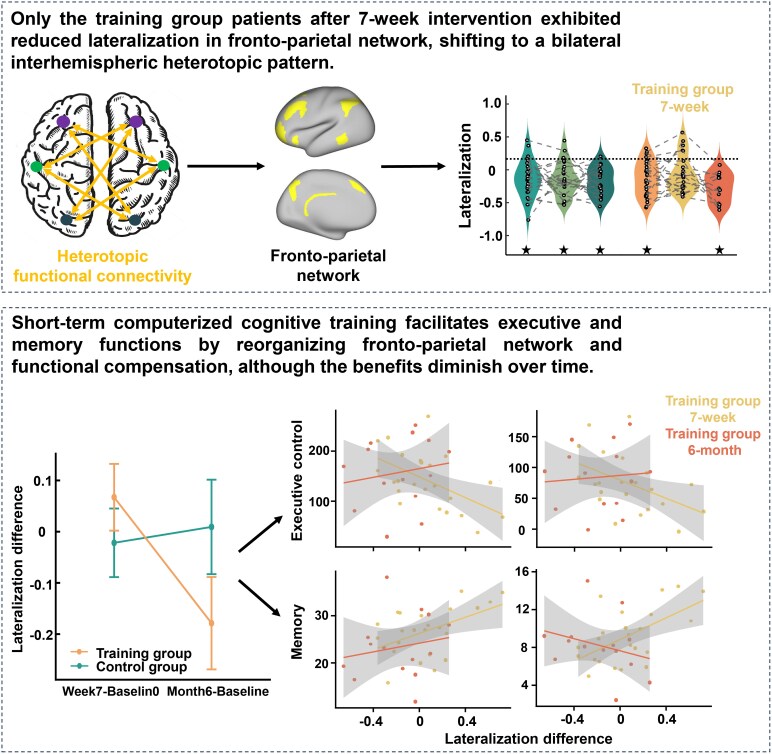

## Introduction

Cognitive decline is common in the elderly, with some developing pathological conditions like dementia.^1^ Mild cognitive impairment marks a critical stage for dementia prevention and treatment. In China, vascular cognitive impairment no dementia (VCIND) is the predominant form of mild cognitive impairment, constituting 42% of cases.^2^ VCIND is characterized by cognitive deficits linked to vascular causes, without meeting dementia diagnosis criteria.^3^ Early intervention of VCIND could delay or reverse cognitive impairment.^4^ Subcortical VCIND, often caused by subcortical ischemic small vessel disease, presents a uniform profile, making it an ideal subject for intervention trials.^5^ However, no approved interventions exist for VCIND. Cognitive training, a structured intervention targeting specific cognitive functions, has been shown to improve general cognitive function in subcortical VCIND patients,^5^ but the underlying brain mechanisms remain underexplored.

This study investigated the mechanisms through brain functional lateralization, a fundamental organizational feature gauging differences between the two hemispheres.^6^ In healthy young adults, language processing is typically left-lateralized, while visuospatial tasks favour the right hemisphere.^6^ However, in older adults, lateralization decreases and bilateral activation increases during tasks. The Hemispheric Asymmetry Reduction in Older Adults (HAROLD) and Scaffolding Theory of Aging and Cognition (STAC) models interpret reduced lateralization in high-performing seniors as compensatory.^7,8^ The effectiveness of this compensation depends on the complementary roles of additionally engaged regions. In some cases, maintaining or enhancing lateralization correlates with improved performance in older adults.^9^ Hence, linking functional lateralization to behavioural performance is essential to differentiate compensation from other mechanisms like neural inefficiency, dedifferentiation, or pathology.^10^ Additionally, the recruitment of right hemisphere regions, not typically support language processing, is common in aphasia recovery after a left-hemisphere stroke.^11^ Stroke research has demonstrated a correlation between the severity of behavioural impairment following focal neural damage and changes in activation and connectivity in remote regions.^12,13^ These findings imply that compensation by reorganization in patients may not be constrained by the intrinsic functions of recruited regions but by structural or functional connectomics.^14^

Neurodegenerative diseases like Alzheimer’s disease could affect brain structural and functional lateralization, even at its pre-dementia stage, such as mild cognitive impairment.^15,16^ However, there has been no reported evidence of lateralization changes in subcortical VCIND, nor the cognitive training effect on lateralization in subcortical VCIND. Previous studies indicate that cognitive training improves executive function and episodic memory in healthy seniors and mild cognitive impairment patients^17,18^ and processes closely linked to the fronto-parietal network (FPN).^19,20^ A meta-analysis shows that cognitive training boosts activation in left FPN regions and reduces it in right frontal regions in healthy older adults, without changes elsewhere,^21^ suggesting that FPN functional lateralization particularly responds to cognitive training. Similarly, cognitive training retains the intrinsic FPN lateralization in older adults, unlike in a control group.^22^ In our prior work using the same dataset of this study, we uncovered increased resting-state functional connectivity (rsFC) between the left dorsolateral prefrontal cortex (a FPN region) and medial prefrontal cortex (a default mode network region) in subcortical VCIND patients after a 7-week cognitive intervention, accompanied by improved Montreal Cognitive Assessment (MoCA) score.^5^ Given that executive dysfunction and memory deficits are associated with VCIND,^23,24^ FPN plays a critical role in cognitive control and episodic memory,^19,20^ and it is sensitive to cognitive training,^5,21,22^ which may improve executive function and episodic memory in subcortical VCIND patients by modulating FPN functional lateralization.

This resting-state functional magnetic resonance imaging (rs-fMRI) study aimed to investigate FPN lateralization in subcortical VCIND patients compared to healthy older adults and how multidomain cognitive training influences executive function and episodic memory by altering FPN lateralization. The primary outcome measures were the trail making test (TMT) for executive function, the auditory verbal learning test (AVLT) for episodic memory and the MoCA for global cognitive function. We defined two functional lateralization indices (LIs) based on rsFC^25-29^: LI of interhemispheric heterotopic FC (LI_he), denoting the left–right difference of FC strength across hemispheres, and LI of intrahemispheric FC (LI_intra), representing the left–right difference of FC strength within the same hemisphere. Our hypotheses were 2-fold. First, compared to healthy older controls, subcortical VCIND patients might exhibit a distinct FPN lateralization pattern or varying correlations between FPN lateralization and task performance that rely on FPN functioning. Second, we aimed to test two alternative hypotheses regarding cognitive training’s effect on FPN lateralization. According to the compensation hypothesis, cognitive training would reduce FPN lateralization, correlating with better task performance. Alternatively, the complementary hypothesis predicts that the training effects on FPN lateralization depend on whether the opposite regions are complementary to task performance. Specifically, reduced lateralization in FPN may not benefit TMT, which involves right-lateralized visual search and working memory,^30^ but may improve AVLT, which involves both left-lateralized verbal processes and right-lateralized memory retrieval.^31^

## Materials and methods

### Clinical trial design and registration

The randomized, active-controlled clinical trial for VCIND patients adhered to the Consolidated Standards of Reporting Trials (CONSORT) statement for clinical and non-pharmacological interventions.^32^ Participants were recruited from Xuanwu, Fu Xing and Beijing Friendship Hospitals of Capital Medical University. The trial was registered at ClinicalTrials.gov (NCT02640716), and its protocol was published previously.^4^

### Participants

A total of 212 individuals from neurology and geriatric clinics were assessed for eligibility. Subcortical VCIND diagnosis was based on cognitive impairment without dementia and small vessel ischemic disease, confirmed by a consensus panel of three senior neurologists. The diagnostic criteria included (i) the main complaint, or reports from an informant, indicated cognitive impairment; (ii) vascular factors covered vascular risk factors, history of stroke, focal neurological signs and imaging evidence of cerebrovascular disease assessed in accordance with version 2 of the Standards for Reporting Vascular Changes on Neuroimaging criteria (STRIVE-2)^33^; (iii) a causal relationship between cognitive impairment and vascular factors was established; (iv) daily functioning was overall intact, though complex instrumental daily ability could be slightly damaged and (v) the criteria of dementia were not met. Inclusion and exclusion criteria are detailed in the Supplementary material and prior publications using the same participants.^4,5^ Finally, 60 subcortical VCIND patients and 30 healthy older adults from the community (healthy group, HG) matched in age and education, but without cognitive impairment complaints or scoring zero on the clinical dementia rating, were recruited. All right-handed participants provided written informed consent. Ethical approval was obtained from the Ethics Committee of Xuanwu Hospital, Capital Medical University.

**Figure 1:**
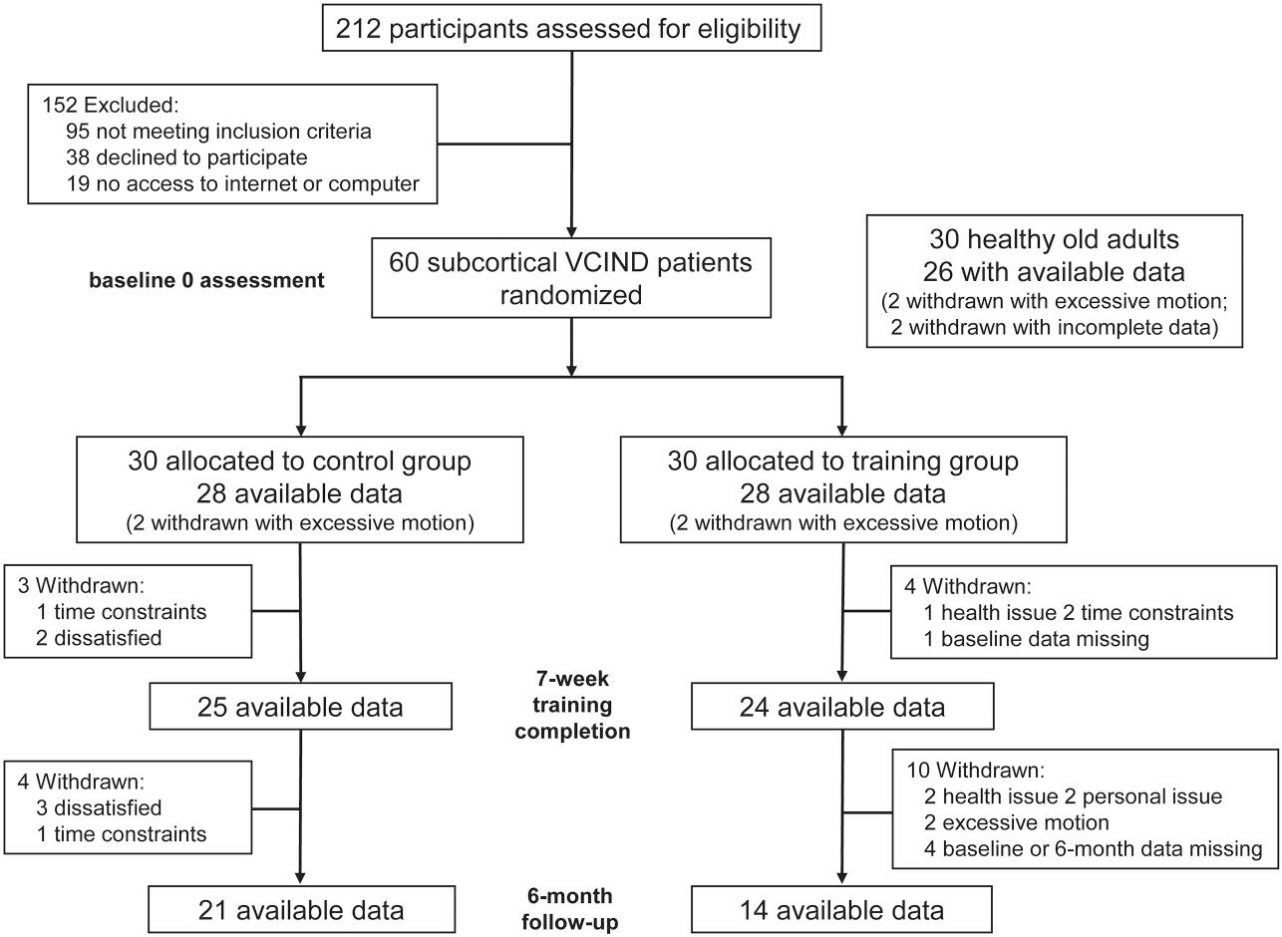
Flowchart for participants’ recruitment, intervention and assessment in this study.

Subcortical VCIND patients were randomly assigned to the training group (TG, *n* = 30) or control group (CG, *n* = 30). The personnel involved in conducting the study and data analysis were masked to the patient randomization. Study participants, their caregivers and all assessors were blinded to treatment assignment throughout the study. The TG received a computerized, adaptive, multidomain training programme targeting processing speed, attention, perception, calculation, long-term memory, working memory, executive control, problem-solving and reasoning. In contrast, the CG performed five fixed, low-difficulty tasks focused on processing speed and attention. Participants in both groups completed 30-min daily training sessions at home, supervised by a neurologist via an online platform (www.66nao.com). The full intervention period for both groups lasted 7 weeks. For the intervention procedure details, see the Supplementary material and our published article.^4^ MRI and behavioural data of HG were acquired only at baseline, while those of patients were collected at Baseline 0, post-intervention (Week 7) and 6-month follow-up (Month 6). Cases with missing data were excluded from analyses for each time point. Of the 82 participants with baseline data (28 TG, 28 CG and 26 HG), 49 (24 TG, 25 CG) finished the intervention and 35 (14 TG, 21 CG) completed the follow-up with available data. Participant flow is detailed in Fig. 1.

### Neuropsychological assessments

We adopted three primary tests: TMT for executive function,^34^ WHO-UCLA AVLT for episodic memory^35^ and MoCA for global cognitive function.^36^ Secondary outcomes included Boston naming test (BNT) and digit span.^37,38^ In our previous work, 7 weeks of cognitive training significantly improved performance only in MoCA and BNT, but not in other measures.^5^

### MRI data acquisition and processing

MRI was performed on a 3T Siemens scanner (Magnetom Skyra). Rs-fMRI was measured using echo-planar imaging with repetition time (TR) = 2000 ms, echo time (TE) = 30 ms, field of view (FOV) = 224 mm, flip angle (FA) = 77°, voxel size = 3.5 × 3.5 × 3.5 mm^3^, 35 slices, lasting 6 min (180 whole-brain volumes). A high-resolution T1-weighted anatomical image was obtained using MP-RAGE (TR = 2100 ms, TE = 2.26 ms, FOV = 256 mm, FA = 12°, slice thickness = 1 mm).

Processing was performed using fMRIPrep 20.2.5^39^ and eXtensible Connectivity Pipeline (XCP-D).^40^ Steps included slice-timing, motion and distortion correction, co-registration to structural data, MNI space normalization and cortical surface projection. Data were subjected to demeaning, detrending and nuisance regression. Volumes with framewise displacement (FD) >0.5 mm—∼8.9% of data—were excluded from nuisance regression. Images were denoised using a 36-parameter confound regression model to minimize motion artifacts.^41^ Residuals were band-pass filtered (0.01–0.08 Hz). Participants with excessive motion or incomplete data were excluded, leaving 82 participants (28 in TG, 28 in CG and 26 in HG) at baseline.

### Calculation of LI and LI difference of FPN

Based on functional homotopy, a core feature of intrinsic brain architecture that has been validated in subcortical vascular cognitive impairment,^42^ we adopted a multimodal parcellation scheme of 360 areas (180 per hemisphere), suitable for assessing FC asymmetries across homologous parcels^43^ at the surface level. The rsFC between two parcels was computed using Pearson correlation (*r*) of BOLD time series, followed by Fisher's *r*-to-*z* transformation.

**Figure 2:**
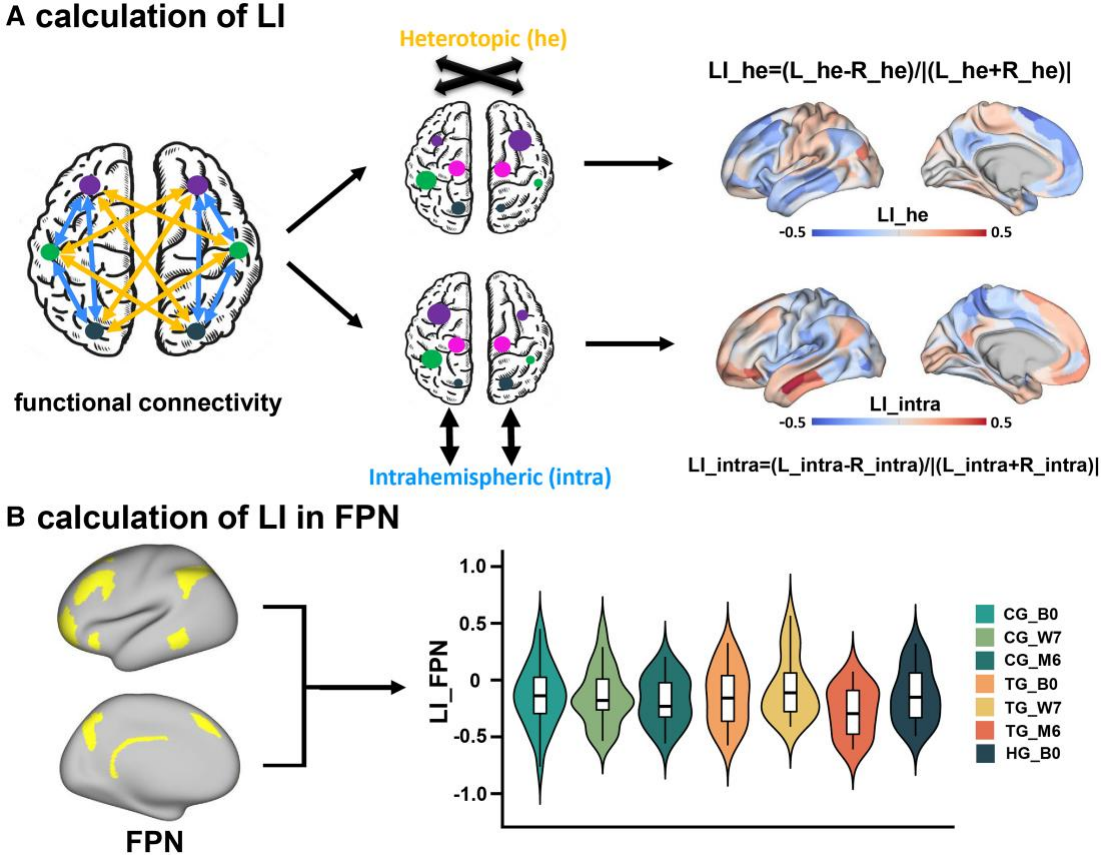
**Workflow of analyses.** (**A**) Calculation of LI. We first defined two different types of functional connectivity (FC) among the whole human brain, named interhemispheric heterotopic FC and intrahemispheric FC. For a specific parcel from the multimodal parcellation scheme of 360 areas (180 per hemisphere), the heterotopic (he) was defined as the sum of heterotopic FCs between this parcel and all others in the opposite hemisphere except the homotopic one, whereas the intrahemispheric (intra) was defined as the sum of intrahemispheric FCs between this parcel and all others within the same hemisphere. The functional lateralization between each homotopic pair of parcels was quantified by laterality index (LI): LI=(L − R)/|(L+R)|, generating two LI maps of LI_he and LI_intra. Larger positive values of LI_he and LI_intra imply stronger across-hemispheric interactions or within-hemispheric interactions in left-hemispheric parcels, respectively, whereas larger negative values indicate stronger interactions in right-hemispheric parcels. (**B**) Calculation of LI in frontoparietal network (FPN). We averaged LIs of homotopic parcels both belonging to FPN and acquired the LI of FPN in control/training group patients and healthy group older adults. CG/TG/HG_B0, control/training/healthy group at baseline 0; CG/TG_W7, control/training group after the 7-week intervention; CG/TG_M6, control/training group at the 6-month follow-up.

For a pair of homotopic parcels, interhemispheric heterotopic LI (LI_he) and intrahemispheric LI (LI_intra), validated by previous studies,^25,26^ were derived as:


(1) $$\mathrm{LI}\_\mathrm{he}=\frac{\left(he_{L}-he_{R}\right)}{\left|he_{L}+he_{R}\right|}$$

(2) $$\mathrm{LI}\_\mathrm{intra}=\frac{\left(\mathrm{intr}a_{L}-\mathrm{intr}a_{R}\right)}{\left|\mathrm{intr}a_{L}+\mathrm{intr}a_{R}\right|}$$

where he was defined as the sum of heterotopic FCs between each parcel and all others in the opposite hemisphere except the homotopic one, while intra denotes the sum of intrahemispheric FCs between each parcel and all others within the same hemisphere (Fig. 2A).

LIs were computed for homotopic FPN parcels from the CAB-NP v1.0 atlas,^44^ validated in elderly and clinical populations.^45,46^ Only considering homotopic parcels both belonging to the FPN, LIs of FPN were calculated by averaging LIs across FPN parcels (Fig. 2B). Larger positive values of LI_he/LI_intra of FPN imply stronger interhemispheric or ipsilateral interactions in left-hemispheric FPN parcels, whereas negative values indicate stronger interactions in right-hemispheric ones. LI differences of FPN were obtained from the same patient by subtracting baseline values from post-intervention measures at Week 7 (CG/TG: *n* = 25/24) and Month 6 (CG/TG: *n* = 21/14).

### Statistical analysis

Group differences in demographic and neuropsychological data were assessed by one-way analyses of variance (ANOVAs), one-way analyses of covariance (ANCOVAs) and independent two-sample *t*-tests. Sex differences were tested by *χ*^2^-tests. LIs were compared amongst groups using one-way ANCOVAs, and *t*-tests identified lateralization patterns for each group.

Training effects on LIs were analysed using linear mixed-effect models, with group (control/training), time (Week 7/Month 6) and group-by-time as fixed effects, and participant as the repeated variable, with age, sex, education, mean FD (mFD) and mean whole-brain rsFCs (mFC) as covariates.^47-51^ As exploratory analyses, partial correlations between LIs or LI differences of FPN and neuropsychological scores were performed, regressing the same confounding variables. Significance was set at *P* < 0.05 after FDR correction (three comparisons) or Games–Howell correction (in cases of heterogeneity of variance) for multiple comparisons. If an effect reached nominal significance (uncorrected *P* < 0.05) but did not survive FDR correction, the uncorrected *P-*value was reported.

## Results

### Baseline neuropsychological performance

Baseline characteristics and neuropsychological assessments are shown in Table 1. We found no group difference in age, sex, education, or neuropsychological scores between the two VCIND groups. However, HG and VCIND groups differed in sex distribution (*χ*^2^ = 11.084, *P* = 0.004, Cramer’s *V* = 0.368), but not age or education. After controlling for sex, significant group differences emerged between HG and VCIND groups in all neuropsychological scores (all *F* > 4.338, *P* < 0.05, *η_p_*^2^ > 0.100) except TMT B-A (*F*(2,79) = 1.333, *P* = 0.270, *η_p_*^2^ = 0.033) and BNT (*F*(2,79) = 2.452, *P* = 0.093, *η_p_*^2^ = 0.059). Thus, subcortical VCIND patients exhibited impaired general cognition, executive function (TMT-B), episodic and working memory compared to healthy older adults.

**Table 1:** Group mean ± standard deviation values, range and statistics of demographic and behavioural data at Baseline 0 in each group

	Control group (CG, *n* = 28)	Training group (TG, *n* = 28)	Healthy group (HG, *n* = 26)	Group difference between CG/TG	Effect size	Group difference amongst CG/TG/HG	Effect size
	Mean ± standard (range)			*t*/*χ*^2^ (*P*)	Cohen’s *d*/Cramer’s *V*	*F*/*χ*^2^ (*P*)	*η_p_* ^2^ */Cramer’s V*
Age (year)	65.32 ± 6.62 (50–78)	65.68 ± 8.02 (48–78)	62.65 ± 6.11 (53–74)	−0.182^a^ (0.856)	−0.049	1.493^c^ (0.231)	0.036
Sex (F/M)	8/20	12/16	19/7	1.244^b^ (0.265)	0.149	11.084^b^ (0.004)	0.368
Education (year)	10.11 ± 2.82 (6–17)	10.61 ± 3.45 (6–17)	11.42 ± 2.87 (6–17)	−0.594^a^ (0.555)	−0.159	1.261^c^ (0.289)	0.031
MoCA (score)	21.25 ± 3.76 (14.0–26.0)	21.79 ± 3.88 (13.0–27.0)	26.69 ± 1.76 (24.0–30.0)	−0.525^a^ (0.602)	−0.140	23.865^d^ (<0.001)	0.299
TMT A (second)	76.92 ± 33.50 (25.5–150.0)	83.60 ± 43.22 (32.9–178.6)	42.51 ± 12.84 (24.4–75.0)	−0.646^a^ (0.521)	−0.173	13.147^d^ (<0.001)	0.187
TMT B (second)	151.31 ± 78.63 (68.1–300.0)	161.21 ± 86.56 (46.0–300.0)	83.33 ± 52.35 (35.0–277.0)	−0.448^a^ (0.656)	−0.120	7.728^d^ (<0.001)	0.115
TMT B-A (second)	74.40 ± 64.99 (−16.4–245.0)	77.61 ± 56.86 (11.5–188.3)	40.82 ± 46.75 (−5.0–218.9)	−0.197^a^ (0.845)	−0.053	1.333^e^ (0.270)	0.033
AVLT_Immediate recall (number)	19.54 ± 6.64 (8.0–31.0)	22.79 ± 6.01 (13.0–34.0)	29.12 ± 4.76 (16.0–38.0)	−1.920^a^ (0.060)	−0.513	13.329^d^ (<0.001)	0.236
AVLT_Delayed recall (number)	6.50 ± 3.16 (1.0–12.0)	7.29 ± 2.77 (1.0–13.0)	11.12 ± 2.72 (5.0–15.0)	−0.989^a^ (0.327)	−0.264	13.966^e^ (<0.001)	0.264
AVLT_Recognition (number)	10.07 ± 2.84 (5.0–14.0)	11.07 ± 2.75 (7.0–15.0)	13.12 ± 1.84 (8.0–15.0)	−1.339^a^ (0.186)	−0.358	6.014^d^ (<0.001)	0.119
Digit span forward	7.18 ± 1.12 (5.0–9.0)	7.25 ± 1.53 (5.0–10.0)	8.46 ± 0.86 (7.0–10.0)	−0.199^a^ (0.843)	−0.053	6.748^e^ (0.002)	0.148
Digit span backward	4.25 ± 1.01 (2.0–6.0)	4.25 ± 1.51 (2.0–8.0)	5.23 ± 1.48 (3.0–8.0)	0.000^a^ (1.000)	0.000	4.338^e^ (0.016)	0.100
BNT	23.61 ± 3.52 (17.0–30.0)	22.00 ± 3.83 (15.0–29.0)	24.85 ± 5.56 (22.0–30.0)	1.635^a^ (0.108)	0.437	2.452^e^ (0.093)	0.059

MoCA, Montreal cognitive assessment; TMT, trail making test; AVLT, WHO-UCLA auditory verbal learning test; BNT, Boston naming test. ^a^Independent two-sample *t*-test. ^b^*χ*^2^-test. ^c^One-way analysis of variance with *F-*value. ^d^One-way analysis of covariance with *F*-value using heteroskedasticity-consistent (HC3) standard errors. ^e^One-way analysis of covariance with *F-*value.

### FPN lateralization in healthy older adults and subcortical VCIND patients

**Figure 3:**
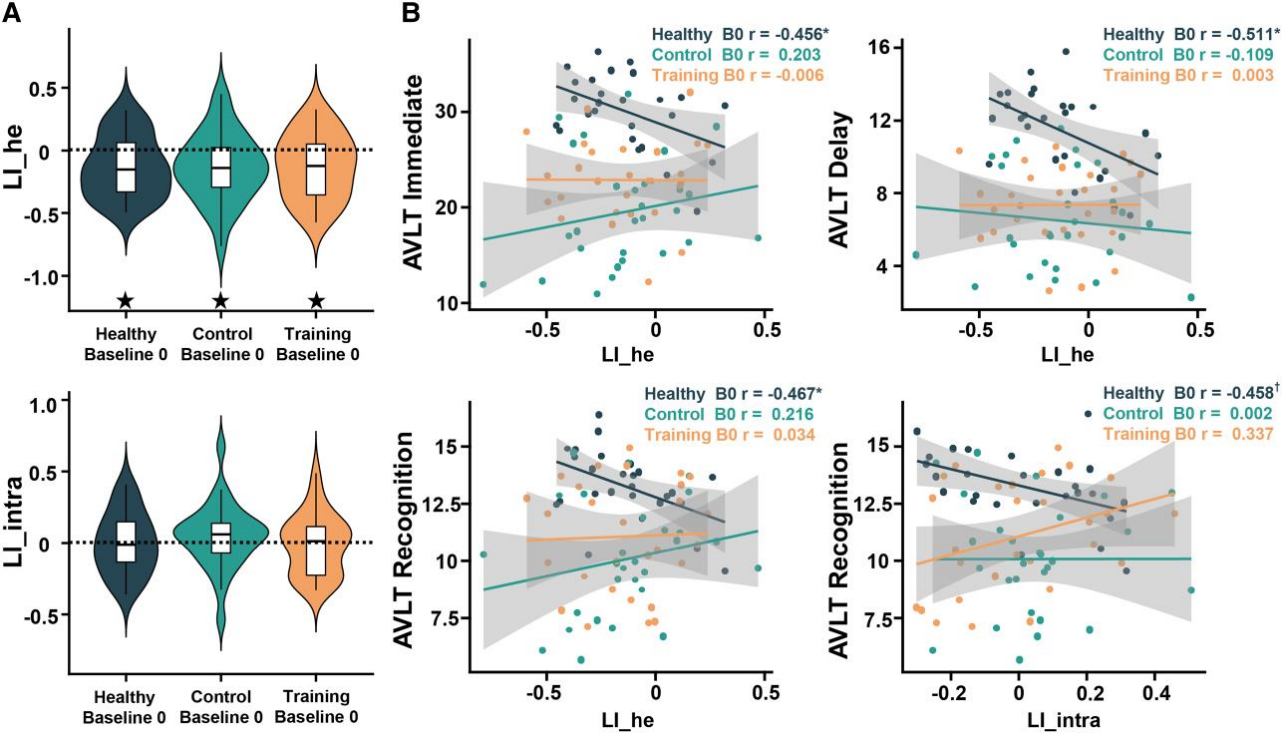
**FPN lateralization patterns and their relationships with behaviours at baseline 0 in healthy group (*n* = 26), control group (*n* = 28) and training group (*n* = 28).** (**A**) LI_he and LI_intra of FPN in three groups at baseline 0. ^★^ FDR-corrected *P* < 0.05 by one-sample *t*-tests. Dashed lines indicate zero (no functional lateralization). (**B**) Partial correlations between LI_he/LI_intra of FPN and AVLT performances (immediate recall, delayed recall, recognition) in three groups at baseline 0. Every dot represents a participant. * FDR-corrected *P* < 0.05, † uncorrected *P* < 0.05. FPN, fronto-parietal network; LI_he, interhemispheric heterotopic LI; LI_intra, intrahemispheric LI.

To reveal how subcortical VCIND changes FPN lateralization, we first explored baseline FPN lateralization patterns. We hypothesized that VCIND patients may exhibit disparate FPN lateralization patterns or changed relationships between FPN lateralization and behavioural performances in executive and memory tasks. Since no group difference existed for mFD (*F*(2,79) = 0.670, *P* = 0.515, *η_p_*^2^ = 0.017) and mFC (*F*(2,79) = 0.471, *P* = 0.626, *η_p_*^2^ = 0.012) amongst three groups, one-way ANCOVAs only controlling for sex revealed no significant group difference in LI_he (*F*(2,79) = 0.003, *P* = 0.997, *η_p_*^2^ < 0.001) or LI_intra (*F*(2,79) = 0.383, *P* = 0.683, *η_p_*^2^ = 0.010). This indicates similar FPN lateralization patterns in healthy older adults and subcortical VCIND patients. All groups exhibited significantly right-lateralized LI_he (all *t* < −2.615, all Cohen’s *d* < −0.494, FDR-corrected *P* = 0.014), but LI_intra was bilateral (Fig. 3A).

We then asked whether and how FPN lateralization was related to neuropsychological performances. Partial correlations revealed significant negative correlations between LI_he and AVLT scores only in HG (immediate recall: *r* = −0.456, FDR-corrected *P* = 0.038; delayed recall: *r* = −0.511, FDR-corrected *P* = 0.038; recognition: *r* = −0.467, FDR-corrected *P* = 0.038, Fig. 3B), suggesting that more right-lateralized LI_he in FPN was linked to better memory in healthy older adults. For LI_intra, more right-lateralized FPN was negatively correlated with better AVLT recognition only in HG (*r* = −0.458, uncorrected *P* = 0.037, FDR-corrected *P* = 0.073). No significant correlations were found in other groups or tasks.

### Effects of cognitive training on FPN lateralization

**Figure 4:**
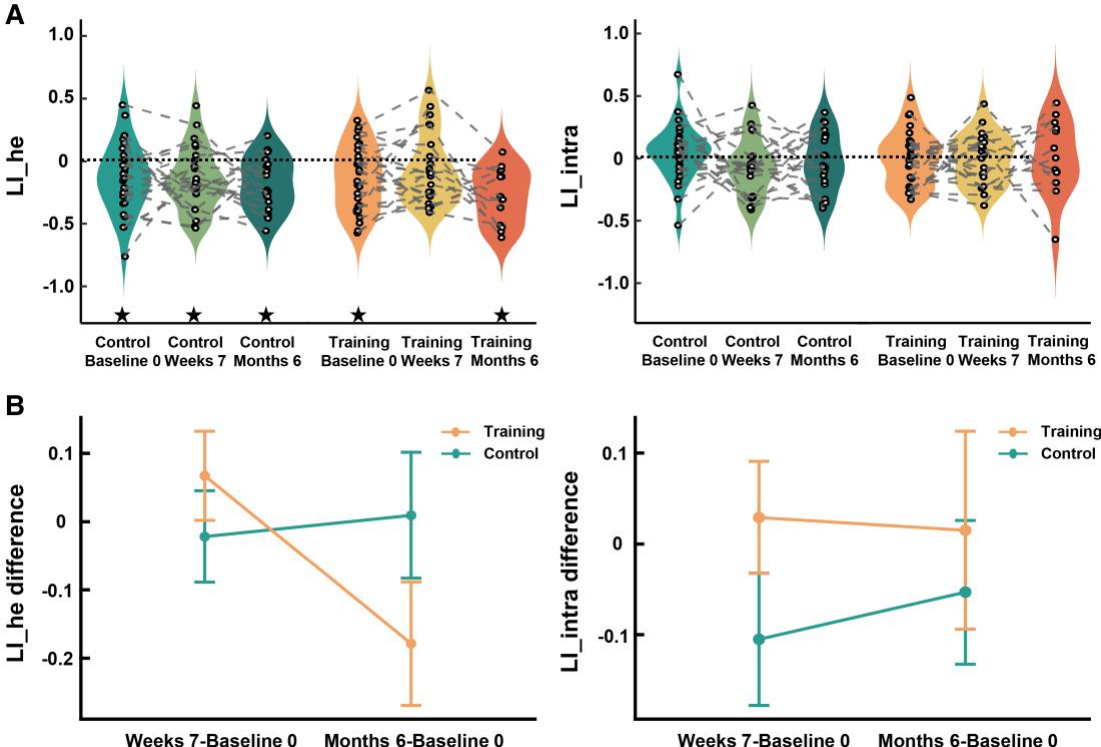
**LIs and LI differences of FPN in subcortical VCIND patients.** (**A**) LI_he and LI_intra of FPN in control group (Baseline 0: *N* = 28; Weeks 7: *N* = 25; Month 6: *N* = 21) and training group (Baseline 0: *N* = 28; Weeks 7: *N* = 24; Month 6: *N* = 14). Every dot represents the LI_he/LI_intra of a participant from control or training group at a certain time point. Values of the same participants among three time points were linked by dashed line. ★ FDR-corrected *P* < 0.05 by one-sample *t*-tests. (**B**) The group (control group/training group) × time (weeks 7—baseline 0/months 6—baseline 0) interactions in LI_he/LI_intra difference of FPN. LI differences of FPN were obtained from the same patient by subtracting baseline values from post-intervention measures at weeks 7 (control/training group: *N* = 25/24) and months 6 (control/training group: *N* = 21/14). Linear mixed-effect models revealed a significant group × time interaction for LI_he difference (*F*(1, 34.90) = 4.571, *P* = 0.040). FPN: fronto-parietal network; LI_he: interhemispheric heterotopic LI; LI_intra: intrahemispheric LI.

To uncover training effects, we compared LI_he and LI_intra of FPN across three time points (Fig. 4A). We expected to observe a training-induced reduction in FPN lateralization. One-sample *t*-tests detected significant right-lateralized LI_he (all *t* < −2.615, all Cohen’s *d* < −0.494, FDR-corrected *P* < 0.05) at all time points in both groups, except for TG at the end of intervention (*t* = −1.194, *P* = 0.245, Cohen’s *d* = −0.244). LI_intra of FPN showed no significant lateralization in either group (−1.526 < *t* < 1.041, *P* > 0.05, −0.305 < Cohen’s *d* < 0.197). Linear mixed-effects models revealed no main effect (0.034 < *F* < 3.008, *P* > 0.05), but a significant group × time interaction for the LI_he difference (*F*(1,34.90) = 4.571, *η_p_*^2^ = 0.120, *P* = 0.040, Fig. 4B), although *post hoc* analyses revealed no significant differences between groups or times (−0.733 < *t* < 2.626, *P* > 0.05). This suggests a shift from right-lateralization to bilateralization in FPN in TG after the 7-week intervention. However, this change did not persist at the 6-month follow-up, moving to a more right-lateralized pattern instead.

### Training effects on neuropsychological scores

**Figure 5:**
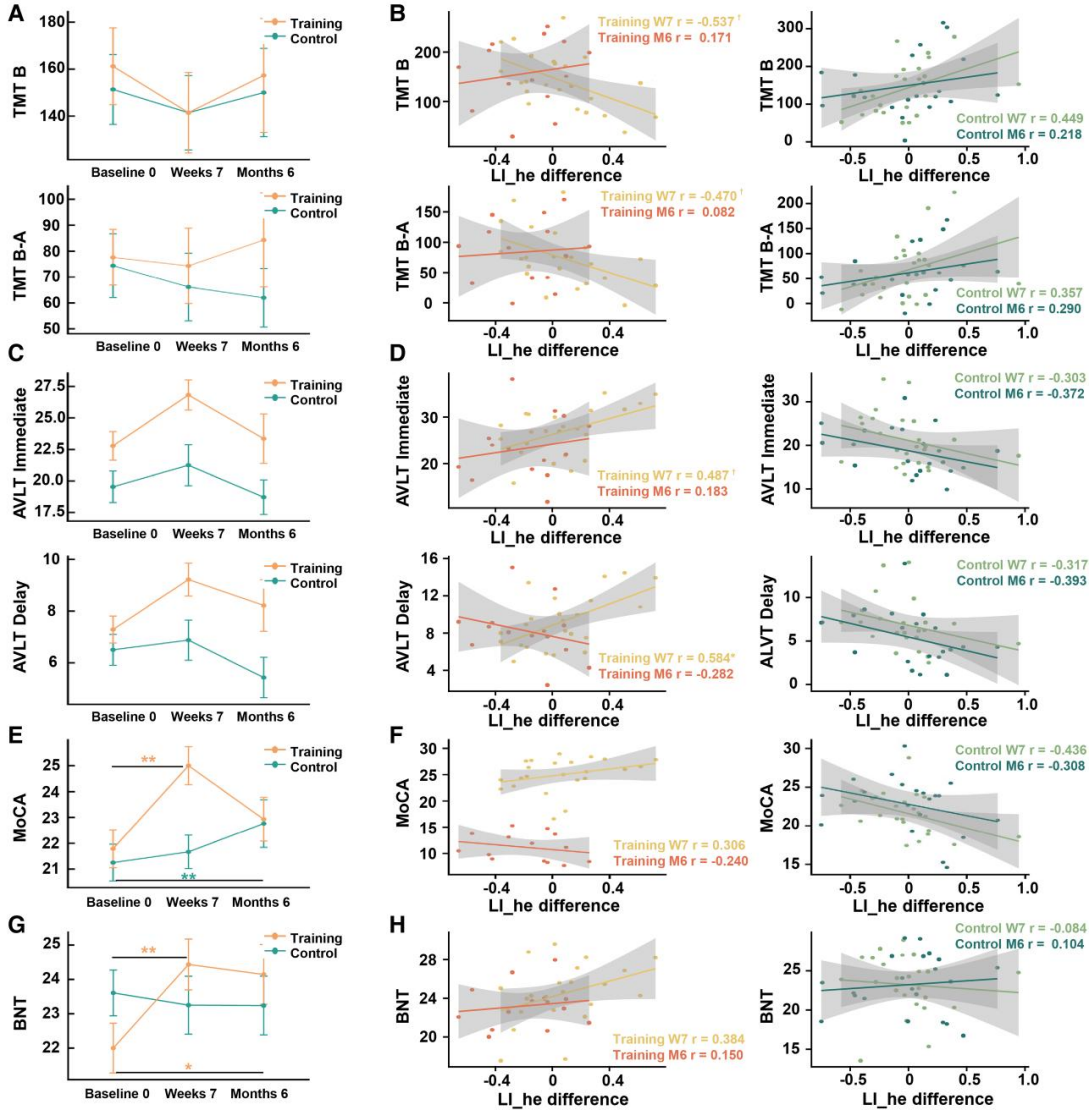
**Behavioural performances and correlations between LI differences of FPN and behaviours in subcortical VCIND patients.** (**A**, **C**, **E**, **G**) Behavioural performances at three time points in training group and control group. **P < 0.01, *P < 0.05, marking significant group × time effects. Partial correlations between LI_he differences of FPN and (**B**) TMT, (**D**) AVLT, (**F**) MoCA and (**H**) BNT performances in training group and control group. Training W7: LI difference of FPN between Week 7 and Baseline 0 in training group (*n* = 24); Training M6: LI difference of FPN between Week 7 and Month 6 in training group (*n* = 14); Control W7: LI difference of FPN between Week 7 and Baseline 0 in control group (*n* = 25); Control M6: LI difference of FPN between Week 7 and Month 6 in control group (*n* = 21). Every dot represents a participant. * FDR-corrected *P* < 0.05, † uncorrected *P* < 0.05. FPN: fronto-parietal network; LI_he: interhemispheric heterotopic LI; LI_intra: intrahemispheric LI.

Linear mixed-effects models including age, sex and education as covariates revealed a significant main effect of time (*F*(2,82.41) = 15.715, *P* < 0.001) and a group (CG/TG) × time (Baseline 0/Week 7/Month 6) interaction (*F*(2,80.79) = 7.264, *η_p_*^2^ = 0.150, *P* = 0.001, Fig. 5E) for MoCA. TG exhibited significant MoCA improvement after 7-week training (*t*(80.5) = 5.895, *P* < 0.001), whereas CG improved MoCA significantly after 6 months (*t*(82.3) = 3.562, *P* = 0.008). Similarly, a significant main effect of time (*F*(2,80.88) = 4.758, *P* = 0.011) and a group (CG/TG) × time (Baseline 0/Week 7/Month 6) interaction (*F*(2,79.14) = 7.687, *η_p_*^2^ = 0.160, *P* < 0.001, Fig. 5G) were found for BNT. BNT significantly improved in TG after 7 weeks (*t*(80.1) = 4.549, *P* < 0.001) and 6 months (*t*(84.2) = 3.389, *P* = 0.013). No training effects were observed for TMT, AVLT or digit span (all *F* < 2.846, *P* > 0.064, Fig. 5A and C).

### The link between FPN lateralization changes and cognitive performances

We then explored whether the change of LI_he in FPN after intervention was linked to better cognitive functions. Partial correlations revealed significant correlations only in TG at the end of the 7-week intervention, but not at the 6-month follow-up. Stronger left-lateralized change of LI_he in FPN was correlated with shorter TMT reaction times (Fig. 5B, TMT B: *r* = −0.537, uncorrected *P* = 0.022, FDR-corrected *P* = 0.065; TMT B-A: *r* = −0.470, uncorrected *P* = 0.049, FDR-corrected *P* = 0.074) and higher AVLT scores (Fig. 5D, immediate recall: *r* = 0.487, uncorrected *P* = 0.040, FDR-corrected *P* = 0.061; delayed recall: *r* = 0.584, uncorrected *P* = 0.011, FDR-corrected *P* = 0.033) in TG at Week 7. These findings indicate that greater left-lateralized changes of FPN lateralization after cognitive training were linked to better executive and memory functions of subcortical VCIND patients. No significant correlations were found for MoCA (Fig. 5F, −0.437 < *r* < 0.307, *P* > 0.05), BNT (Fig. 5H, −0.085 < *r* < 0.385, *P* > 0.05) or digit span (−0.116 < *r* < 0.248, *P* > 0.05).

## Discussion

From an unexplored perspective of intrinsic functional lateralization of brain networks, this study provides new insights that short-term multidomain computerized cognitive training reorganizes the functional lateralization of FPN in subcortical VCIND patients. Specifically, the intervention shifted the FPN lateralization from a right-lateralized pattern to bilateral symmetry, which improves patients’ executive and memory functions. Before training, both patients and healthy older adults exhibited comparable right-lateralized interhemispheric FC within the FPN. However, a stronger right-lateralized LI_he in FPN correlated with better memory performance solely in the healthy group, suggesting that subcortical VCIND disrupts the functional relationship between FPN lateralization and behavioural performance rather than the lateralization itself. After 7 weeks of training, the FPN right lateralization shifted towards a bilaterally symmetric pattern in the VCIND training group. Importantly, greater leftward changes in FPN lateralization were linked with better TMT and AVLT performance after the intervention, underscoring the compensatory role of functional reorganization in FPN. In contrast, no changes were observed in the control group or at the 6-month follow-up, indicating that sustained cognitive training is necessary to maintain these benefits.

These findings align with compensation theories, suggesting that short-term cognitive training mitigates cognitive deficits by reorganizing the FPN functional lateralization rather than reinstating their original configurations in subcortical VCIND patients, which benefits FPN-supported executive and memory functions. Our study offers new insights into the potential and mechanism of computerized cognitive training as a therapeutic intervention for cognitive impairments.

### Dysfunction of FPN lateralization in subcortical VCIND

FPN plays a central role in cognitive control, which supports a range of cognitive functions, including working memory, encoding and retrieval during episodic memory.^20^ In young adults, the interhemispheric connections from the right intraparietal sulcus/frontal eye field to the left counterpart exhibit greater strength than those in the opposite direction, suggesting a right-lateralized interhemispheric connectivity within the FPN.^52^ This FPN lateralization pattern may contribute to cognitive reserve in healthy older adults resistant to neurodegeneration in mild cognitive impairment and Alzheimer’s disease. Cognitive reserve refers to the phenomenon where older adults who engage in intellectually stimulating environments experience less decline in cognitive abilities and are better able to maintain cognitive functions despite the presence of degenerative brain changes associated with Alzheimer’s disease.^53^ Previous studies have shown that connectivity in the right FPN is crucial for preserved alertness function amongst healthy older participants,^54^ and higher levels of cognitive reserve in older adults are associated with increased involvement of the right FPN during visual information processing.^55^ Considering that cognitive reserve develops through repeated activation of norepinephrine with its privileged association with the right FPN underlying arousal, sustained attention, working memory, self-monitoring processes and novelty, a prominent role for the right FPN in cognitive reserve is proposed.^30^

Consistent with this idea, this study observed a significant correlation between right-lateralized LI_he in FPN and better AVLT performance amongst healthy older adults. A little to our surprise, we found no difference in the lateralization pattern of FPN between the healthy group and the VCIND groups. However, the significant correlations between LI_he of FPN and AVLT scores in healthy older adults were absent in patients with subcortical VCIND. This may be explained by diaschisis, a well-established phenomenon describing the temporary interruption of function in brain regions remote from the injured site, particularly following stroke.^14^ Studies in stroke patients have discovered that the severity of behavioural impairment often correlates with altered activation and connectivity in regions distant from the lesion,^12,13^ which disrupts normal functional lateralization. These findings indicate that ‘functional deafferentation’ of remote areas can lead to abnormal functional lateralization that is sufficient to impair cognitive performance. Consistent with findings in stroke patients, our results suggest that subcortical VCIND does not completely disrupt the lateralization pattern of the FPN, but instead selectively impairs the functioning of the right-lateralized FPN in maintaining normal episodic memory.

### Reorganization of FPN lateralization through cognitive training

Cognitive training can effectively improve cognitive functions in subcortical VCIND patients.^5^ While evidence demonstrates its ability to mitigate aging-related dysfunction in higher cognitive networks,^56,57^ only our previous study using the same dataset reported increased FC between the dorsolateral prefrontal cortex and medial prefrontal cortex following short-term cognitive training in subcortical VCIND patients.^5^ The underlying brain mechanisms and timing of changes after training remain unclear. Here, we examined functional lateralization, a fundamental intrinsic organization feature, to explore how cognitive training influences FPN lateralization and its association with cognitive functions in subcortical VCIND patients.

We found significantly reduced interhemispheric FC lateralization in FPN, shifting from right-lateralized to bilaterally symmetric, exclusively in the VCIND training group. This multidomain training targeted FPN-supported functions, including flexibility, problem solving, long-term memory, working memory, processing speed and attention. In contrast, the control group, which only received attention and processing speed tasks, showed no significant changes in FPN lateralization. These findings suggest that targeted engagement of FPN-supported cognitive processes may drive network-level plasticity and promote more balanced hemispheric involvement. Notably, these changes were observed immediately after the intervention but not at the 6-month follow-up, consistent with our previous findings that the 7-week training period was insufficient for long-term effects.^5^

Consistent with the compensation hypothesis, alterations in FPN lateralization after intervention were linked to improved performance on TMT (TMT B/TMT B-A) and AVLT (immediate recall/delayed recall) in the VCIND training group. Specifically, greater changes towards bilateral or even left-lateralized FPN were associated with better executive and memory functions, supporting that cognitive training alleviates these prominent impairments in subcortical VCIND^23,58^ by rebalancing interhemispheric FPN lateralization. This reorganization likely reflects compensatory neural recruitment from additional regions, a mechanism often observed in older adults^14^ and stroke recovery.^59,60^ The extent of focal neural damage and the severity of behavioural impairment in stroke patients correlate with compensatory recruitment,^59^ altered functional connectivity,^60^ and greater functional reorganization^61^ in remote areas. Thus, the correlation between a more bilateral or left-lateralized FPN and better performance in the current study aligns with findings in stroke recovery, as well as the HAROLD^7^ and the STAC models in aging research,^8^ and extends these theories of compensation by reorganization to subcortical VCIND patients following cognitive training. Furthermore, the absence of brain-behaviour correlation at the 6-month follow-up indicates that continuous cognitive training is highly recommended for patients with subcortical VCIND to sustain cognitive improvements.

### Limitations and future investigation

Our findings provide evidence that short-term cognitive training rebalances FPN lateralization, offering a compensatory mechanism for cognitive deficits in subcortical VCIND. These changes suggest that FPN functional lateralization may serve as a potential biomarker for evaluating intervention outcomes and brain plasticity. However, several limitations warrant consideration in future studies. Some results did not survive correction for multiple comparisons and showed wide confidence intervals; nonetheless, the observed correlations and group differences were substantial enough to support our findings. Given that the intervention effects appeared after 7 weeks but did not persist at the 6-month follow-up, a longer intervention period as well as assessments at intermediate time points may be required to achieve lasting benefits and to better understand the temporal dynamics of the intervention and its underlying brain mechanisms. Although we excluded individuals with indications of hippocampus or entorhinal atrophy to reduce the likelihood of mixed pathology, such as concomitant Alzheimer’s disease, we could not rule out the presence of prodromal Alzheimer’s disease with increased amyloid load. Future studies incorporating Alzheimer’s disease biomarkers are needed to rigorously assess the efficacy of cognitive training in patients with subcortical VCIND and to determine whether distinct underlying pathologies differentially influence the response to cognitive training. Larger sample sizes and inclusion of well-matched healthy older adults are needed to validate these findings, especially considering participant attrition during the 6-month follow-up. Moreover, while repetitive neuropsychological testing may have induced learning effects, these did not appear to impact between-group differences or the group × time interaction. Finally, whether other interventions (e.g. physical exercise, pharmacological treatments) can produce similar changes in PFN lateralization remains an open question and warrants future investigation.

In summary, from a previously unidentified perspective of resting-state functional lateralization, we found that (i) patients with subcortical VCIND exhibited a right-lateralized FPN similar to healthy older adults, but this right lateralization pattern did not support memory function as it did in healthy older adults; (ii) via compensation by reorganizing the functional lateralization of FPN from right-lateralized to bilaterally symmetric, the computerized, adaptive, multidomain cognitive training was beneficial to executive and memory functions in patients with subcortical VCIND after intervention. Thus, healthy older adults and patients with subcortical VCIND may rely on different coping strategies and distinct functional lateralization patterns of FPN to achieve better executive and memory performance. This helps deepen the understanding of the influence of subcortical VCIND on the brain and inspires cognitive training intervention. The correlations between the functional lateralization of FPN and cognitive abilities make it a potential biomarker for evaluating the intervention outcomes and underlying brain plasticity. Although the efficacy and good safety profile of computerized cognitive training recommend its application in patients with subcortical VCIND, more clinical trials are needed for further evidence.

## Supplementary Material

fcaf394_Supplementary_Data

## Data Availability

The datasets used and/or analysed during the current study are available from the corresponding author on reasonable request. Data were analysed using the codes uploaded on GitHub (https://github.com/Xinhu-Jin/VCIND/tree/main/script).
