# Supplementary material for: Cognitive training reorganizes lateralization of fronto-parietal network in vascular cognitive impairment
Source: Brain Commun. 2025 Oct 10;7(6):fcaf394. doi: 10.1093/braincomms/fcaf394 (PMC12607259; doi:10.1093/braincomms/fcaf394)
Supplement: fcaf394_Supplementary_Data [file fcaf394_supplementary_data.docx]

**Supplementary material**

**Materials and methods**

Participants

Inclusion and exclusion criteria

The diagnosis of VCIND was based on evidence of both cognitive impairment without dementia and small vessel ischemic disease. All patients were diagnosed by a consensus panel including three senior neurologists and met the following inclusion criteria: 1. literate in Han Chinese with a consistent caregiver (> 4 days/week); 2. complaint and/or informant report of cognitive impairment involving memory and/or other cognitive domains with a duration of at least 3 months; 3. according to the Diagnostic and Statistical Manual of Mental Disorders, Fourth Edition,^1^ the patients were neither normal nor demented as indicated by a clinical dementia rating of ≥ 0.5 on at least one domain, a global score of ≤ 0.5, and a Mini–Mental State Examination score of ≥ 20 (primary school) or ≥ 24 (junior school or above); 4. normal or slightly impaired daily living activities as defined by a total score of ≤ 1.5 for the three functional clinical dementia rating domains (home and hobbies, community affairs, and personal care). We excluded participants who exhibited any condition that would preclude completion of neuropsychological testing (e.g., deafness, aphasia) or disorders other than subcortical VCIND that would affect cognition (e.g., Alzheimer’s disease, dementia with Lewy Bodies).

The MRI-based inclusion criteria details were as follows: 1. multiple (≥ 3) supratentorial subcortical small infarcts (3–20 mm in diameter) with/without white matter lesions of any degree or moderate-to-severe white matter lesions (score of ≥ 2 according to the Fazekas rating scale^2^) with/without small infarct; 2. absence of cortical or watershed infarcts, hemorrhages, hydrocephalus, or white matter lesions with specific causes (e.g., multiple sclerosis); 3. no hippocampal or entorhinal cortex atrophy (score of zero according to the medial temporal lobe atrophy scale of Scheltens^3^).

Exclusion criteria included the following: 1. severe aphasia or other factors that might preclude completion of neuropsychological assessments or MRI; 2. clinically significant gastrointestinal, renal, hepatic, respiratory, or other systemic diseases; 3. other disorders or use of medication that might affect cognitive functions.

Intervention procedure

As multi-domain training protocol has been approved as effective approach for cognitive training in mild cognitive impairment population^4,5^ and healthy elderly,^6^ the present study has designed a multi-domain training protocol. The training tasks were from domains of processing speed, perception, attention, long-term memory, working memory, calculation, executive control, reasoning and problem solving. Specific training paradigms included number and object detection, letter and chess selection, dual-attending task, attention span task, face-name paired-association task, one-back and n-back working memory task, Go-Nogo task, Stroop task, Flanker task and switching task. The rigor with which each domain was trained differed according to each task and informed the grouping of the tasks. Participants were required to complete 30 min of training per day (five 2-min tasks completed thrice), five days a week. To enable adaptive training, each task was designed with several difficulty levels. Based on previous tests with a large size sample, the tasks will be further grouped in each domain with varied task difficulty. At the beginning, assignment tasks from these domains will be similar across participants. On each training day, five tasks (2 min per task, each three times, in total 30 min per day) will be assigned. Within each task, high accuracy (> 80 %) is required to upgrade. To manipulate the adaptive change, the number of types of stimuli, the presentation probability of each type of stimuli, and the size and duration of a stimulus were systematically set. To keep a systematical setting, only one parameter will be changed, while the other parameters will be kept as constant in one level upgraded. Once the task performance is higher than 80 % of the norm performance of a normal aging population, the task will be replaced by a harder task from the same domain. The training is thus also adaptive at participant level, with a similar setup but personalized progress across participants.

The active control group received five processing speed and attention tasks, whose duration totaled 30 min each training day. However, these tasks were set to a fixed, primary difficulty level across the study. The training of all participants was completed at home and supervised by an independent neurologist over the Internet (www.66nao.com) to guarantee the fulfillment of the training. Specifically, the neurologist supervised daily if the participants finished the training and the training time through the Internet. If a certain participant missed the training, the neurologist would contact the participant and his/her family to remind him/her to accomplish the training.

**References:**

1. American Psychiatric Association A, American Psychiatric Association. *Diagnostic and Statistical Manual of Mental Disorders: DSM-IV*. Vol 4. American psychiatric association Washington, DC; 1994.

2. Fazekas F, Chawluk J, Alavi A, Hurtig H, Zimmerman R. MR signal abnormalities at 1.5 T in Alzheimer’s dementia and normal aging. *American Journal of Roentgenology*. 1987;149(2):351-356. doi:10.2214/ajr.149.2.351

3. Scheltens P, Leys D, Barkhof F, et al. Atrophy of medial temporal lobes on MRI in" probable" Alzheimer’s disease and normal ageing: diagnostic value and neuropsychological correlates. *Journal of Neurology, Neurosurgery & Psychiatry*. 1992;55(10):967-972.

4. Hill NT, Mowszowski L, Naismith SL, Chadwick VL, Valenzuela M, Lampit A. Computerized cognitive training in older adults with mild cognitive impairment or dementia: a systematic review and meta-analysis. *American Journal of Psychiatry*. 2017;174(4):329-340.

5. Sherman DS, Mauser J, Nuno M, Sherzai D. The Efficacy of Cognitive Intervention in Mild Cognitive Impairment (MCI): a Meta-Analysis of Outcomes on Neuropsychological Measures. *Neuropsychol Rev*. 2017;27(4):440-484. doi:10.1007/s11065-017-9363-3

6. Lampit A, Hallock H, Valenzuela M. Computerized Cognitive Training in Cognitively Healthy Older Adults: A Systematic Review and Meta-Analysis of Effect Modifiers. Gandy S, ed. *PLoS Med*. 2014;11(11):e1001756. doi:10.1371/journal.pmed.1001756
